# Optimal trial design selection: a comparative analysis between two-arm and three-arm trials incorporating network meta-analysis for evaluating a new treatment

**DOI:** 10.1186/s12874-023-02089-y

**Published:** 2023-11-11

**Authors:** Fangshu Ye, Chong Wang, Annette M. O’Connor

**Affiliations:** 1 Department of Statistics, College of Liberal Arts and Sciences, Iowa State University, Ames, IA USA; 2 Department of Veterinary Diagnostic and Production Animal Medicine, College of Veterinary Medicine, Iowa State University, Ames, IA USA; 3 Department of Large Animal Clinical Sciences, College of Veterinary Medicine, Michigan State University, East Lansing, MI USA

**Keywords:** Network meta-analysis, Clinical trial design, Evidence synthesis

## Abstract

**Background:**

Planning the design of a new trial comparing two treatments already in a network of trials with an a-priori plan to estimate the effect size using a network meta-analysis increases power or reduces the sample size requirements. However, when the comparison of interest is between a treatment already in the existing network (old treatment) and a treatment that hasn’t been studied previously (new treatment), the impact of leveraging information from the existing network to inform trial design has not been extensively investigated. We aim to identify the most powerful trial design for a comparison of interest between an old treatment A and a new treatment Z, given a fixed total sample size. We consider three possible designs: a two-arm trial between A and Z (’direct two-arm’), a two-arm trial between another old treatment B and Z (’indirect two-arm’), and a three-arm trial among A, B, and Z.

**Methods:**

We compare the standard error of the estimated effect size between treatments A and Z for each of the three trial designs using formulas. For continuous outcomes, the direct two-arm trial always has the largest power, while for a binary outcome, the minimum variances among the three trial designs are conclusive only when $p_A(1-p_A) \ge p_B(1-p_B)$. Simulation studies are conducted to demonstrate the potential for the indirect two-arm and three-arm trials to outperform the direct two-arm trial in terms of power under the condition of $p_A(1-p_A) < p_B(1-p_B)$.

**Results:**

Based on the simulation results, we observe that the indirect two-arm and three-arm trials have the potential to be more powerful than a direct two-arm trial only when $p_A(1-p_A) < p_B(1-p_B)$. This power advantage is influenced by various factors, including the risk of the three treatments, the total sample size, and the standard error of the estimated effect size from the existing network meta-analysis.

**Conclusions:**

The standard two-arm trial design between two treatments in the comparison of interest may not always be the most powerful design. Utilizing information from the existing network meta-analysis, incorporating an additional old treatment into the trial design through an indirect two-arm trial or a three-arm trial can increase power.

## Background

Network meta-analysis (NMA) compares three or more interventions by combining indirect and direct evidence from a network of trials. When designing a new trial, NMA can be used to leverage existing trial data, reducing the sample size needed and increasing the power to detect treatment effects [1].

Nikolakopoulo et al. (2014) [2] provided a framework for study design that helps investigators decide the treatments, total sample sizes, and the number of studies needed to achieve a desirable level of power, with the existing evidence. While their study examined the comparison of interest (COI) between treatments that existed in the network of trials, an important and valuable situation is to compare one treatment that appears in the existing network of trials and one treatment that doesn’t. We refer to the treatment that appeared previously and the treatment that didn’t appear previously as ’old treatment’ and ’new treatment’, respectively. We are unaware of any guidance or literature investigating the study design for a future trial when the COI is between a new treatment and an old treatment.

Suppose a specific total sample size has been decided based on the available physical or financial resources. When a specific comparison (AZ) is of interest with A as an old treatment and Z as a new treatment, it is of interest to know which of the possible designs provides the greatest power. The most intuitive design is to conduct a two-arm trial between A and Z directly. Rigorous evidence is needed to validate the intuition. When we analyze the new trial with the existing evidence from a network of trials, it is possible that we can gain power when indirect evidence is introduced. Another motivation to consider other types of design is that the old treatment A in our interested COI is expensive or practically hard to implement. For example, perhaps treatment A is an antibiotic with a longer withholding period compared to treatment B, so although legal and feasible, for a trial it would not be preferred by the farm staff. Another rationale could be that treatment B is already used at the planned trial site, and implementing two novel treatments (A and Z) at the trial site is a barrier to the conduct of the trial In such situations, we would actually look for alternative designs by including another relatively old treatment to avoid the higher resource cost associated with A but still be able to provide a reliable estimate of the relative effect size between A and Z at the same time. As a consequence of these motivations, researchers could be interested in exploring the power among three possible trial designs: 1) direct two-arm trial: conduct a new two-arm trial between A and Z; 2) three-arm trial: conduct a new three-arm trial among A, B and Z; 3) indirect two-arm trial: conduct a new two-arm trial between B and Z where B is another old treatment.

Our aim is to provide guidelines for investigators to decide the most powerful trial design among the three candidates. We develop formulas for both continuous and binary outcomes and investigate if borrowing information from the existing evidence can increase power. The three trial designs are compared based on their maximum achievable power under a fixed total sample size. Sample size allocation will be optimized to minimize the variance, thereby maximizing the power. To evaluate the power difference further, a simulation study is conducted to illustrate the power difference among the three candidate trial designs. By doing so, we hope to provide valuable insights into designing future trials and facilitating the efficient use of existing resources.

## Methods

In this section, we introduce the variance formula for the three designs under two types of outcome data, continuous and binary. The comparison of power among the three designs is achieved by comparing the variance of the estimated effect size.

The following set of notations are used for the two types of outcomes. Suppose our COI is between treatments A and Z, where A is an old treatment and Z is a new treatment. Treatment B is another old treatment in the network. Let $d_{AZ, two}$, $d_{AZ, two, indirect}$, and $d_{AZ, three}$ denote the relative effect size between treatment A and Z in the direct two-arm trial, indirect two-arm trial and three-arm trial, respectively. Let $\hat{d}_{AZ, two}$ , $\hat{d}_{AZ, two, indirect}$ , and $\hat{d}_{AZ, three}$ denote the corresponding estimations. Let $n_i$ denote the sample size for each treatment group *i*, $i\in \{A,B,Z\}$. We use $\sigma ^2_{AB,old}$ to denote the variance of the estimated effect size between treatment A and B from the existing NMA.

### Continuous outcome

Assume we have a two-arm trial comparing treatment A and Z with a total sample size of *n*. Suppose the outcome data are continuous such as a production metric like average daily gain or milk production. In the continuous case, we use the mean difference in the outcome to represent the relative effect size. The variance of $\hat{d}_{AZ,two}$ can be written as

$$\begin{aligned} \text {Var}(\hat{d}_{AZ,two})=\hat{\sigma }^2_{AZ}\times \left( \frac{1}{n_A}+\frac{1}{n_Z}\right) \end{aligned}$$

where $\sigma ^2_{AZ}$ is the variance of response for each treatment group under the homogeneous variance assumption and $\hat{\sigma }^2_{AZ}$ is the estimate. The optimal allocation would be $n_A=n_Z=\frac{n}{2}$, then the minimal value of $\text {Var}(\hat{d}_{AZ,two})$ is

1 $$\begin{aligned} \min (\text {Var}(\hat{d}_{AZ,two})) = \frac{4\hat{\sigma }^2_{AZ}}{n} \end{aligned}$$

Suppose instead we conduct a two-arm trial with treatment B and Z with a total sample size of *n*, and the comparison between treatment A and Z can be achieved by using the indirect estimate obtained from adding the new trial data to the existing network using NMA. The variance of $\hat{d}_{AZ, two, indirect}$ can be expressed as

$$\begin{aligned} \text {Var}(\hat{d}_{AZ,two,indirect})=\hat{\sigma }^2_{BZ}\times \left( \frac{1}{n_B}+\frac{1}{n_Z}\right) + \sigma ^2_{AB, old} \end{aligned}$$

where $\sigma ^2_{BZ}$ is the variance of response for each treatment group under the homogeneous variance assumption and $\hat{\sigma }^2_{BZ}$ is the estimate. $\text {Var}(\hat{d}_{AZ,two, indirect})$ reaches its minimum when $n_B = n_Z = \frac{n}{2}$ and its minimum is

2 $$\begin{aligned} \min (\text {Var}(\hat{d}_{AZ,two,indirect}))= \frac{4\hat{\sigma }^2_{BZ}}{n} + \sigma ^2_{AB, old} \end{aligned}$$

Finally, suppose we conduct a three-arm trial with treatments A, B and Z with a total sample size of *n*, the variance of $\hat{d}_{AZ, three}$ when we analyze the new trial with the existing network by NMA is

$$\begin{aligned} \text {Var}(\hat{d}_{AZ,three}) = \hat{\sigma }^2\left[ \frac{1}{n_A} + \frac{1}{n_Z} -\frac{1}{n_A^2\left(\frac{\hat{\sigma }_{AB,old}^2}{\hat{\sigma }^2}+\frac{1}{n_A}+\frac{1}{n_B}\right)}\right] \end{aligned}$$

where $\sigma ^2$ is the variance of response for each treatment group in the three-arm trial under the homogeneous variance assumption and $\hat{\sigma }^2$ is the estimate.

For any given sample size $n_A$, $n_B$ and $n_Z$, $\text {Var}(\hat{d}_{AZ,three})$ is always bigger than

$$\begin{aligned} \text {Var}(\hat{d}_{AZ,three,0})= & {} \hat{\sigma }^2\left[ \frac{1}{n_A} + \frac{1}{n_Z} -\frac{1}{n_A^2\left(\frac{1}{n_A}+\frac{1}{n_B}\right)}\right] \\= & {} \hat{\sigma }^2\left[ \frac{1}{n_A} + \frac{1}{n_Z} -\frac{n_B}{n_A(n_A+n_B)}\right] \\= & {} \hat{\sigma }^2\left[ \frac{1}{n_Z} + \frac{n_A+n_B}{n_A(n_A+n_B)} -\frac{n_B}{n_A(n_A+n_B)}\right] \\= & {} \hat{\sigma }^2\left[ \frac{1}{n_Z} + \frac{n_A}{n_A(n_A+n_B)}\right] \\= & {} \hat{\sigma }^2\left[ \frac{1}{n_Z} + \frac{1}{n_A+n_B}\right] \end{aligned}$$

For a fixed total sample size $n = n_A+n_B+n_Z$, $\text {Var}(\hat{d}_{AZ,three,0})$ reaches the minimum when $n_Z = n_A+n_B = \frac{n}{2}$ and the minimal value of $\text {Var}(\hat{d}_{AZ,three,0})$ is

3 $$\begin{aligned} \min (\text {Var}(\hat{d}_{AZ,three,0})) =\hat{\sigma }^2\left[ \frac{2}{n} + \frac{2}{n}\right] = \frac{4\hat{\sigma }^2}{n} \end{aligned}$$

By the homogeneous variance assumption for each treatment group, we have $\sigma _{AZ}^2 = \sigma _{BZ}^2 = \sigma ^2$ so that $\hat{\sigma }_{AZ}^2 = \hat{\sigma }_{BZ}^2 = \hat{\sigma }^2$. By Eqs. 1, 2, and 3, we have the following two inequalities

$$\begin{aligned} \min (\text {Var}(\hat{d}_{AZ,two,indirect}))= \frac{4\hat{\sigma }^2_{BZ}}{n} + \sigma ^2_{AB, old} > \frac{4\hat{\sigma }^2_{BZ}}{n} = \frac{4\hat{\sigma }^2_{AZ}}{n} = \min (\text {Var}(\hat{d}_{AZ,two})) \end{aligned}$$

$$\begin{aligned} \min (\text {Var}(\hat{d}_{AZ,three})) > \min (\text {Var}(\hat{d}_{AZ,three,0})) = \frac{4\hat{\sigma }^2}{n} = \frac{4\hat{\sigma }^2_{AZ}}{n} = \min (\text {Var}(\hat{d}_{AZ,two})) \end{aligned}$$

To summarize, we have

$$\begin{aligned} \min (\text {Var}(\hat{d}_{AZ,two,indirect}))> \min (\text {Var}(\hat{d}_{AZ,two})); \qquad \min (\text {Var}(\hat{d}_{AZ,three}))> \min (\text {Var}(\hat{d}_{AZ,two})) \end{aligned}$$

Given the total sample size is fixed at *n*, the minimum variance of the estimated effect size between treatment A and Z of the direct two-arm trial is the smallest among the three types of design. In other words, it’s unnecessary to conduct a three-arm trial or indirect two-arm trial in the continuous case for the purpose of reducing variance or increasing power. This result is independent of the configuration of the network of trials i.e. the number of trials for each treatment or the effect size of any pairwise comparison of A, B or Z.

### Binary outcome

Assume we have a two-arm trial comparing treatment A and Z with a total sample size of *n*. Suppose the outcome is binary such as a disease event. For binary data, we usually use the log odds ratio between two groups to represent the relative effect size. Let $p_i$ denote the estimated probability of an event occurring in treatment group *i*, $i\in \{A,B,Z\}$. The variance of $\hat{d}_{AZ,two}$ can be written as

4 $$\begin{aligned} \text {Var}(\hat{d}_{AZ,two})=\frac{1}{n_A p_A (1-p_A)} + \frac{1}{n_Z p_Z (1-p_Z)} \end{aligned}$$

By calculating the first derivative and setting it to 0, the optimal sample size allocation with the goal to minimize $\text {Var}(\hat{d}_{AZ,two})$ would be

$$\begin{aligned} n_Z&= \frac{n}{1+\sqrt{\frac{p_Z(1-p_Z)}{p_A(1-p_A)}}}\\ n_A&= n - n_Z \end{aligned}$$

The minimal value of $\text {Var}(\hat{d}_{AZ,two})$ with a fixed total sample size of *n* is

5 $$\begin{aligned} \min (\text {Var}(\hat{d}_{AZ,two}))= \frac{1}{n}\left[ \frac{1}{p_Z(1-p_Z)} + \frac{1}{p_A(1-p_A)} + 2 \times \sqrt{\frac{1}{p_Zp_A(1-p_Z)(1-p_A)}} \right] \end{aligned}$$

Suppose we conduct a new two-arm trial with treatment B and Z with a total sample size of *n*, the variance of the estimated effect size between treatment A and Z by analyzing the new trial with the existing network by NMA can be expressed as

$$\begin{aligned} \text {Var}(\hat{d}_{AZ,two, indirect})= \frac{1}{n_B p_B (1-p_B)} + \frac{1}{n_Z p_Z (1-p_Z)} + \sigma ^2_{AB, old} \end{aligned}$$

Similar to Eq. 5, we have the minimal value of $\text {Var}(\hat{d}_{AZ,two, indirect})$ to be

6 $$\begin{aligned} \min (\text {Var}(\hat{d}_{AZ,two, indirect}))= \frac{1}{n}\left[ \frac{1}{p_Z(1-p_Z)} + \frac{1}{p_B(1-p_B)} + 2\times \sqrt{\frac{1}{p_Zp_B(1-p_Z)(1-p_B)}}\right] + \sigma ^2_{AB, old} \end{aligned}$$

Suppose we conduct a new three-arm trial with treatment A, B and Z with a total sample size of *n*, the variance of $\hat{d}_{AZ, three}$ when we analyze the new trial with the existing network by NMA is:

7 $$\begin{aligned} \text {Var}(\hat{d}_{AZ,three})) = \frac{1}{n_A p_A (1-p_A)} + \frac{1}{n_Z p_Z (1-p_Z)} - \frac{1}{[n_A p_A (1-p_A)]^2 \left(\sigma ^2_{AB, old}+\frac{1}{n_A p_A (1-p_A)} + \frac{1}{n_B p_B (1-p_B)}\right)}. \end{aligned}$$

To determine if there exists any condition(s) where the indirect or three-arm trial would result in a smaller variance other than the direct two-arm trial (as was the case for the continuous outcomes), we utilize Eqs. (4)-(6). For simplicity, the formulas are re-written as below:

$$\begin{aligned} v_1&\equiv \min (\text {Var}(\hat{d}_{AZ,two})) = \frac{1}{n}\left(\frac{1}{q_Z} + \frac{1}{q_A} + 2\sqrt{\frac{1}{q_Zq_A}}\right)\\ v_2&\equiv \min (\text {Var}(\hat{d}_{AZ,two,indirect})) = \frac{1}{n}\left(\frac{1}{q_Z} + \frac{1}{q_B} + 2\sqrt{\frac{1}{q_Zq_B}}\right) + \sigma ^2_{AB, old} \\ v_3&\equiv \text {Var}(\hat{d}_{AZ,three})) = \frac{1}{n_A q_A} + \frac{1}{n_Z q_Z} - \frac{1}{n_A^2q_A^2 \left(\sigma ^2_{AB, old}+\frac{1}{n_A q_A} + \frac{1}{n_B q_B}\right)} \end{aligned}$$

where $q_i = p_i(1-p_i)$ for $i \in {A, B, Z}$. Let $v_{3,0} = \frac{1}{n_A q_A} + \frac{1}{n_Z q_Z} - \frac{1}{n_A^2q_A^2 (\frac{1}{n_A q_A} + \frac{1}{n_B q_B})}$. It is straightforward to see that $v_3 > v_{3,0}$.

Under the condition that $q_A \ge q_B$, we have the relationship between $v_1$ and $v_2$ as follows:

8 $$\begin{aligned} v_2&> \frac{1}{n}\left(\frac{1}{q_Z} + \frac{1}{q_B} + 2\sqrt{\frac{1}{q_Zq_B}}\right)\nonumber \\&\ge \frac{1}{n}\left(\frac{1}{q_Z} + \frac{1}{q_A} + 2\sqrt{\frac{1}{q_Zq_A}}\right)\nonumber \\&\equiv v_1 \end{aligned}$$

Let $v_{3,0}' = \frac{1}{n_Aq_A} + \frac{1}{n_Zq_Z} - \frac{1}{n_A^2q_A^2 (\frac{1}{n_A q_A} + \frac{1}{n_B q_A})}$. Under the condition that $q_A \ge q_B$, it is obvious that $v_{3,0} \ge v_{3,0}'$. $v_{3,0}'$ can be simplified as

$$\begin{aligned} v_{3,0}'&=\frac{1}{n_Aq_A} + \frac{1}{n_Zq_Z} - \frac{1}{n_Aq_A \left(1+\frac{n_A}{n_B}\right)}\\&=\frac{1}{n_Aq_A} + \frac{1}{n_Zq_Z} - \frac{1}{n_Aq_A \left(\frac{n_A+n_B}{n_B}\right)}\\&=\frac{1}{n_Aq_A} + \frac{1}{n_Zq_Z} - \frac{n_B}{n_Aq_A (n_A+n_B)}\\&=\frac{1}{n_Aq_A}\left(1-\frac{n_B}{n_A+n_B}\right) + \frac{1}{n_Zq_Z}\\&=\frac{1}{n_Aq_A}\cdot \frac{n_A}{n_A+n_B} + \frac{1}{n_Zq_Z}\\&=\frac{1}{q_A(n-n_Z)}+\frac{1}{n_Zq_Z} \\&=\frac{\left(\frac{1}{\sqrt{q_A}}\right)^2}{n-n_Z}+\frac{\left(\frac{1}{\sqrt{q_Z}}\right)^2}{n_Z}. \end{aligned}$$

Sedrakyan’s inequality [3] states that for any reals $a_1, \cdots , a_n$ and positive reals $b_1, \cdots , b_n$, we have $\sum _{i=1}^n\frac{a_i^2}{b_i} \ge \frac{(\sum _{i=1}^n a_i)^2}{\sum _{i=1}^n b_i}$. From the expression of $v_{3,0}'$, we have $a_1 = \frac{1}{\sqrt{q_A}}$, $a_2 = \frac{1}{\sqrt{q_Z}}$, $b_1 = n-n_Z$, and $b_2 = n_Z$, therefore we have

$$\begin{aligned} v_{3,0}'&\ge \frac{1}{n-n_Z+n_Z}\left(\frac{1}{\sqrt{q_A}} + \frac{1}{\sqrt{q_Z}}\right)^2\nonumber \\&= \frac{1}{n}\left(\frac{1}{q_A}+\frac{1}{q_Z}+2\sqrt{\frac{1}{q_A q_Z}}\right)\nonumber \\&\equiv v_1. \end{aligned}$$

With above, we have $v_{3,0}' \ge v_1$, $v_{3,0} \ge v_{3,0}'$, $v_3 > v_{3,0}$. To sum up,

9 $$\begin{aligned} v_3 > v_{3,0} \ge v_{3,0}' \ge v_1 \end{aligned}$$

By Eqs. (8) and (9), we find that $v_2 > v_1$ and $v_3 > v_1$ when $q_A \ge q_B$, which means, under the condition that $p_A(1-p_A) \ge p_B(1-p_B)$, the minimum variance of the estimated effect size between treatment A and Z of the direct two-arm trial is the smallest among all three types of design. Therefore, it is reasonable to choose the direct two-arm trial as the trial design under the condition of $p_A(1-p_A) \ge p_B(1-p_B)$ for the consideration of power.

However, for the scenario where $p_A(1-p_A) < p_B(1-p_B)$, the relationship between $v_1$, $v_2$ and $v_3$ is uncertain, indicating that any of the three types of trial design could have the smallest minimum variance based on different parameters (*n*, $p_A$, $p_B$ and $\sigma ^2_{AB,old}$), which will be shown later by the simulation study. Recall that the minimum variance refers to the minimum variance of the estimated effect size between treatment Z and A that can be achieved by the optimal allocation of total sample size *n*. Each type of trial design with a fixed *n* has its minimum variance and the smallest one among the three minimum variances is called the smallest minimum variance.

### Optimal sample allocation with a fixed total sample size

In Binary outcome section, we show that for each type of design with a fixed total sample size, the minimum variance of the estimated effect size could be achieved by altering the sample size allocation. Some variance formulas like Eq. 7 are complex to calculate a numeric solution to the optimal sample size allocation given a fixed total sample size for minimizing the variance. For other variance formulas like Eq. 4, even though it is straightforward to calculate the optimal sample size allocation for the direct and indirect two-arm trial. It is still one step away from the final solution due to the constraint that the sample size for each treatment group has to be an integer. Considering those factors, we get the optimal sample size allocation with a fixed total sample size and binary outcome by solving the following optimization problems:

For the direct two-arm trial,

10 $$\begin{aligned} \text {minimize}\ \text {Var}(\hat{d}_{AZ,two})=\frac{1}{n_A p_A (1-p_A)} + \frac{1}{n_Z p_Z (1-p_Z)} \end{aligned}$$

$$\begin{aligned} \text {s.t }&n_A + n_Z= n\\ n_A&\text {and}\ n_Z\ge 10\\ n_A&\text {and}\ n_Z\ \text {are positive integers}. \end{aligned}$$

For the indirect two-arm trial,

11 $$\begin{aligned} \text {minimize}\ \text {Var}(\hat{d}_{AZ,two, indirect})= \frac{1}{n_B p_B (1-p_B)} + \frac{1}{n_Z p_Z (1-p_Z)} + \sigma ^2_{AB, old} \end{aligned}$$

$$\begin{aligned} \text {s.t}\ {}{} & {} n_B + n_Z= n\\ n_B{} & {} \text {and}\ n_Z\ge 10\\ n_B{} & {} \text {and}\ n_Z\ \text {are positive integers}. \end{aligned}$$

For the three-arm trial,

12 $$\begin{aligned}&\text {minimize}\ \text {Var}(\hat{d}_{AZ,three})) = \nonumber \\&\frac{1}{n_A p_A (1-p_A)} + \frac{1}{n_Z p_Z (1-p_Z)} - \frac{1}{[n_A p_A (1-p_A)]^2 \left(\sigma ^2_{AB, old}+\frac{1}{n_A p_A (1-p_A)} + \frac{1}{n_B p_B (1-p_B)}\right)} \end{aligned}$$

$$\begin{aligned} \text {s.t}&n_A + n_B + n_Z= n\\ n_A, n_B&\text {and}\ n_Z\ge 10\\ n_A, n_B&\text {and}\ n_Z\ \text {are positive integers}. \end{aligned}$$

We set the constraint on the minimum number of samples for each treatment to be 10 to ensure the statistical inference on the new trial is based on a reasonable number of subjects in each group. Without this constraint, it is possible to have a sample size of 1 mathematically, which is not practically feasible. The constraint value 10 can be changed to other appropriate numbers according to the practical trial scenario. Closed-form solutions do not exist for minimizing this function. Therefore, nonlinear optimization methods are used to obtain the optimal allocation. In particular, we utilize the “differential evolution optimization” [4]. This optimization method searches over a continuous space so the integer solution can be obtained by enumerating all possible integers around the global solution.

### Power formula

In the previous two subsections, we present the variance formula when the outcome type is continuous and binary. The ultimate goal for reducing the variance is to maximize the power. In this section, we present the formula for estimating the probability that the effect size in log odds ratio scale between two treatments will be statistically significant under a specific alternative hypothesis, i.e, power. Let $\mu _{AZ}$ be the true effect size between treatment A and Z. Let the null hypothesis $H_0$ for the comparisons *AZ* be $\mu _{AZ}=0$. Let $\mu _{AB}\ne 0$ be the alternative hypothesis $H_1$, then the expressions for the power is given by

13 $$\begin{aligned} \text {Power}=\Phi \left(\frac{\mu _{AZ}}{s.d}-z_{\alpha /2}\right)+\Phi \left(-\frac{\mu _{AZ}}{s.d}-z_{\alpha /2}\right), \end{aligned}$$

where *s*.*d* denotes the population standard deviation of the effect size $\mu _{AZ}$, which is often replaced with the standard error of the estimated effect size; $\Phi (\cdot )$ is the standard normal cumulative distribution function; $\alpha$ is the significance level; $z_{\alpha /2}$ is the upper $\alpha /2$th quantile of the standard normal distribution, which is used here to control the overall type I error of the testing procedure at level $\alpha$.

## Simulation

### Dataset description

**Fig. 1:**
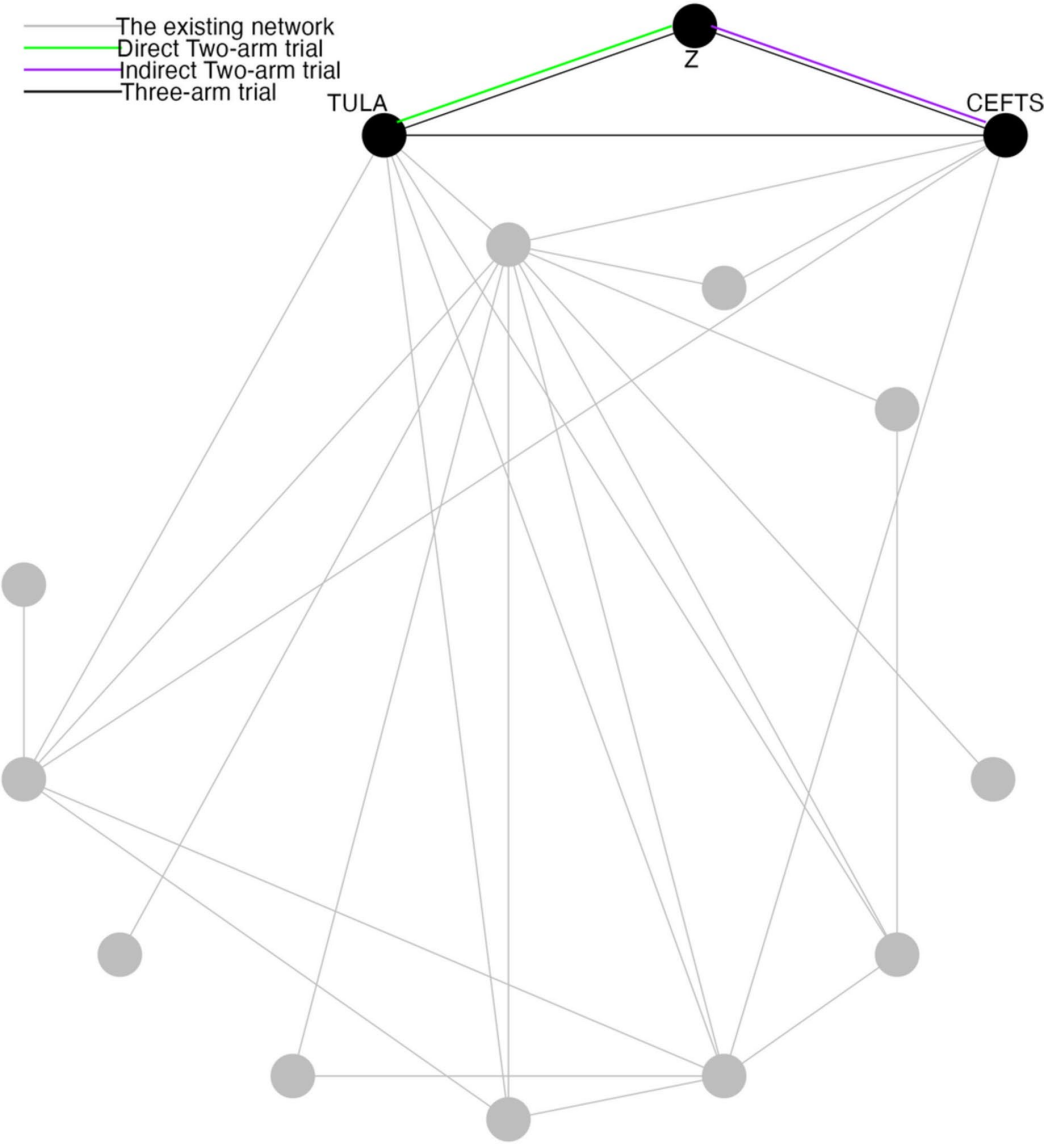
An existing network of interventions for the treatments of Bovine Respiratory Disease (BRD) in feedlot cattle with a new trial that intends to compare tulathromycin (TULA) and a new treatment Z. The new trial has three candidate trial designs including two-arm trial between TULA and Z, two-arm trial between ceftiofur sodium (CEFTS) and Z, three-arm trial among TULA, CEFTS and Z

A previously published network of interventions for the antibiotic treatment of Bovine Respiratory Disease (BRD) in feedlot cattle is used as an illustrative example for the problem of interest [5]. The network comprises 98 trials and 13 treatments in total. Most trials contain two arms and eight trials contain three arms. The network plot is shown in Fig. 1. Arm-level data are available and the outcome is a dichotomous health event. To compare treatments, the log odds ratios for pairwise comparisons are calculated. Our focus for illustration purposes is the antibiotic tulathromycin (TULA) and a combination product of sulfamethoxazole/trimethoprim (TRIM) or ceftiofur sodium (CEFTS). All products are administered according to the manufactures instructions. More details about these data are available in the original publication [5].

### Simulation

In Methods section, the relationship of the minimum variance among three types of design is conclusive if the outcome is continuous. For binary data, the minimum variance among three trial designs is determinable when $p_A(1-p_A) \ge p_B(1-p_B)$ while it is not when $p_A(1-p_A) < p_B(1-p_B)$. To illustrate the possibility for the two alternatives to be the best design regarding power under the condition of $p_A(1-p_A) < p_B(1-p_B)$, two simulation studies with binary outcomes are conducted in this section. Two scenarios are included to illustrate that the power gain is dependent upon the comparison of interest due to different disease risks and the extent of information (prior trials) in the network. The simulations are employed to compare the maximum power that each trial design can achieve given the fixed total sample size. Notably, the key distinction between Simulation I and Simulation II lies in the selection of treatment B for the alternative trial designs. We present these specific simulation scenarios to demonstrate the potential for each alternative to emerge as the optimal trial design among the three candidate options. The selection of treatment B is based on an initial exploration of the power formula, providing valuable insights into the potential advantages of each trial design.

### Simulation I: example scenario for indirect two-arm to be the most powerful trial design

Assume our COI is between treatment TULA and treatment Z and three options of trial design are open to be chosen (Fig. 1). In the three-arm trial, treatment CEFTS is selected from the existing network as the third treatment. For the convenience of notation, we denote TULA and CEFTS as A and B in this subsection. From the NMA of the existing network, the estimated risk of A and B are 0.166 and 0.430, which ensures the condition of $p_A(1-p_A) < p_B(1-p_B)$ holds.

We set the total sample size of the new trial to be 80/100/120 and set 4 different values from 0.35 to 0.50 as the risk of the new treatment, Z. For each scenario with the risk of *Z* to be $p_Z$ and the total sample size *n*, the process is conducted as below:

1. From a network meta-analysis of the existing network, the risk of treatment *j* is estimated and denoted as $p_j$.
2. Analyze the direct two-arm trial between A and Z
  - Find the optimal allocation ($n_A$, $n_Z$) by solving the optimization problems in Eq. 10.
  - Data representing the new trial is generated by sampling $r_i$ from Binom($n_i$, $p_i$), $i \in \{A, Z\}$.
  - Exact logistic regression is applied to analyze the data and the p-value is extracted from the result.
  - Use a 0-1 indicator to denote if there is a significant difference between the A and Z ($\alpha =0.05$).
  - Repeat steps above for 50,000 times. Calculate the proportion of the indicator equal to 1 to obtain the simulation power.
3. Analyze the indirect two-arm trial between B and Z.
  - Find the optimal allocation ($n_B$, $n_Z$) by solving the optimization problems in Eq. 11.
  - Data representing the new trial is generated by sampling $r_i$ from Binom($n_i$, $p_i$), $i \in \{B, Z\}$.
  - The data representing the new trial is added to the existing network to represent a row of study-level data.
  - Network meta-analysis is applied to analyze the combined data and the p-value is extracted from the result.
  - Use a 0-1 indicator to denote if there is a significant difference between A and Z ($\alpha =0.05$).
  - Repeat steps above for 50,000 times. Calculate the proportion of the indicator equal to 1 to get the simulation power.
4. Analyze the three-arm trial with A, B, and Z.
  - Find the optimal allocation ($n_A$, $n_B$, $n_Z$) by solving the optimization problems in Eq. 12.
  - Data representing the new trial is generated by sampling $r_i$ from Binom($n_i$, $p_i$), $i \in \{A, B, Z\}$.
  - The data representing the new trial is added to the existing network to represent a row of study-level data.
  - Network meta-analysis is applied to analyze the combined data and the p-value is extracted from the result.
  - Use a 0-1 indicator to denote if there is a significant difference between A and Z ($\alpha =0.05$).
  - Repeat steps above for 50,000 times. Calculate the proportion of the indicator equal to 1 to get the simulation power.

### Simulation II: example scenario for three-arm to be the most powerful trial design

**Fig. 2:**
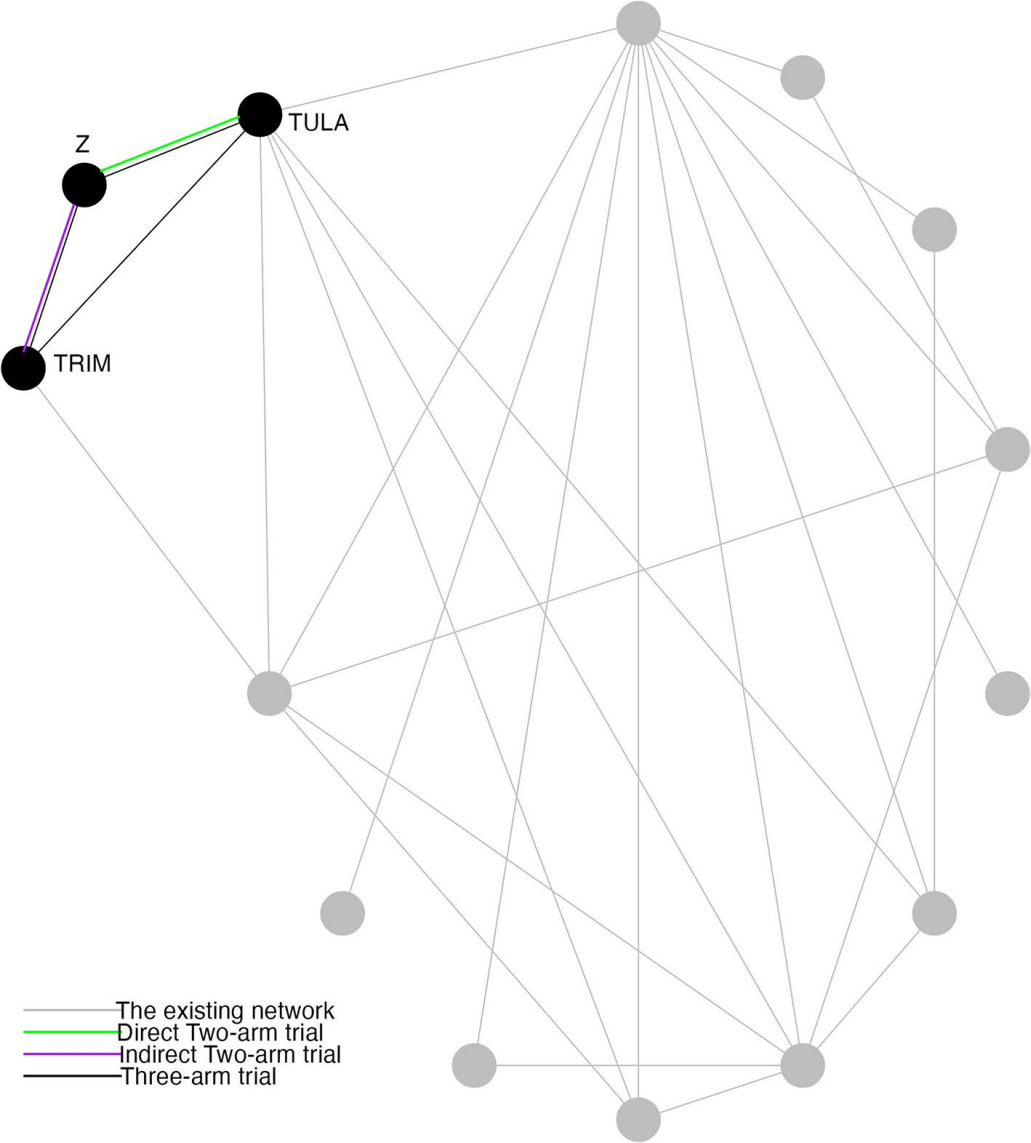
An existing network of interventions for the treatments of Bovine Respiratory Disease (BRD) in feedlot cattle with a new trial that intends to compare tulathromycin (TULA) and a new treatment Z. The new trial has three candidate trial designs including two-arm trial between TULA and Z, two-arm trial between sulfamethoxazole/trimethoprim (TRIM) and Z, three-arm trial among TULA, TRIM and Z

Assume our COI is between treatment TULA and treatment Z. We set 9 values from 0.35 to 0.43 as the risk of the new treatment, Z. For each scenario with the risk of *Z* to be $p_Z$ and the total sample size $n=100$, the process of simulation II is the same as simulation I, except for the choice of treatment B (Fig. 2). In this simulation, treatment A is the same as simulation I while treatment B is TRIM. From the NMA of the existing network, the estimated risk of A and B are 0.166 and 0.553, which satisfies the condition of $p_A(1-p_A) < p_B(1-p_B)$.

## Results

The outputs from Simulation Study I are in Table 1. Each row represents a different scenario with a different risk of Z, $p_Z$, and total sample size, *N*. In each scenario, it records the optimal sample size allocation given a fixed total sample size of a new trial for each trial design from left to right: (1) direct two-arm trial; (2) three-arm trial; (3) indirect two-arm trial. The power of each trial design in each scenario can be found in the same row. The powers of trial design (2) and (3) both surpass that of trial design (1). In other words, a three-arm trial with TULA, CEFTS and the new treatment Z or a two-arm trial with CEFTS and Z is better in power than a two-arm trial with TULA and Z when our COI is between TULA and Z. Take the fifth row for example, when the risk of Z is 0.35 and the total sample size for the new trial is fixed at 100, from simulation, we are able to gain an additional $8.2\%$ power when we select the trial design (2) and an additional $11.8\%$ power when we select the trial design (3) compared with trial design (1).

**Table 1:** Power difference and sample size allocation among three trial settings in Simulation Study I: Treatment A is TULA, Treatment B is CEFTS. The estimated risk of A and B are 0.166 and 0.430 from the existing network meta-analysis

		Sample size allocation in different trial setting							Simulation				
		(1)		(2)			(3)		Power (%)			Power Difference (%)	
Risk of Z	N	Z	A	Z	A	B	Z	B	(1)	(2)	(3)	(2) - (1)	(3) - (1)
0.35	80	35	45	39	10	31	41	39	42.6	50.7	55.5	8.1	12.9
0.40	80	35	45	38	10	32	40	40	60.7	69.5	73.3	8.8	12.6
0.45	80	34	46	38	10	32	40	40	76.5	83.6	86.7	7.1	10.2
0.50	80	34	46	38	10	32	40	40	87.7	92.6	94.4	4.9	6.6
0.35	100	44	56	49	10	41	51	49	52.2	60.5	64.0	8.2	11.8
0.40	100	43	57	48	10	42	50	50	71.2	79.4	81.6	8.2	10.4
0.45	100	43	57	48	10	42	50	50	85.6	91.1	92.6	5.5	7.1
0.50	100	43	57	48	10	42	50	50	94.2	96.9	97.5	2.7	3.3
0.35	120	53	67	59	10	51	61	59	60.3	69.1	71.9	8.9	11.6
0.40	120	52	68	58	10	52	60	60	79.6	86.1	87.9	6.5	8.3
0.45	120	51	69	58	10	52	60	60	91.6	95.2	96.0	3.7	4.4
0.50	120	51	69	58	10	52	60	60	97.3	98.6	98.8	1.3	1.6

The outputs from Simulation Study II are in Table 2. Each row represents a different scenario in Simulation Study II with a different risk of Z, $p_Z$. The left part records the optimal sample size allocation given a fixed total sample size of 100 to maximize the power for each trial design. The power of each trial design in each scenario can be found in the same row. Same as Table 1, The trial design from (1) to (3) represents direct two-arm trial, three-arm trial, and indirect two-arm trial respectively. In Simulation Study II, trial design (2) has the largest power among the three trial designs, which means, conducting a three-arm trial is the best option in terms of power when our COI is between TULA and Z while the other available treatment in the existing network is TRIM. Take the first row for example, when the risk of Z is 0.35 and the optimal allocation is applied for each trial design to reach its best power, trial design (2) increases $2.0\%$ and $3.7\%$ in power simulation-wise compared with trial design (1) and (3).

**Table 2:** Power difference and sample size allocation among three trial settings in Simulation Study II: Treatment A is TULA, Treatment B is TRIM. The estimated risk of A and B are 0.166 and 0.553 from the existing network meta-analysis

		Sample size allocation in different trial setting							Simulation				
		(1)		(2)			(3)		Power (%)			Power Difference (%)	
Risk of Z	N	Z	A	Z	A	B	Z	B	(1)	(2)	(3)	(2) - (1)	(2) - (3)
0.35	100	44	56	46	38	16	51	49	52.2	54.3	50.5	2.0	3.7
0.36	100	44	56	46	38	16	51	49	56.5	58.4	54.8	2.0	3.7
0.37	100	44	56	46	38	16	51	49	60.4	62.6	59.2	2.2	3.4
0.38	100	43	57	46	38	16	51	49	63.9	66.5	63.2	2.6	3.2
0.39	100	43	57	46	38	16	50	50	67.7	70.3	67.5	2.6	2.8
0.40	100	43	57	46	38	16	50	50	71.2	74.0	71.3	2.9	2.8
0.41	100	43	57	45	39	16	50	50	74.5	77.6	74.8	3.1	2.9
0.42	100	43	57	45	39	16	50	50	77.6	80.7	78.0	3.1	2.7
0.43	100	43	57	45	39	16	50	50	80.5	83.5	80.9	3.0	2.6

## Discussion

### Direct two-arm trial is not always the best

Support our COI is between one new treatment (Z) and one old treatment (A) from the existing network, a two-arm trial is commonly the first choice. However, with the network meta-analysis, the possibilities of the trial design are expanded. In this paper, we explore three different types of trial design including direct two-arm trial, indirect two-arm trial, and three-arm trial. In both indirect two-arm trial and three-arm trial, we introduce another old treatment (B) from the existing network to leverage the existing information of the comparison between A and B to inform the comparison between A and Z. From the method part (Binary outcome section), we conclude that when $p_A(1-p_A) \ge p_B(1-p_B)$, the direct two-arm trial between A and Z would always be the best choice in term of power. However, when $p_A(1-p_A) < p_B(1-p_B)$, it is possible to leverage information from NMA to gain additional power by using either indirect two-arm trial between Z and B or the three-arm trial among A, B and Z. As we show in Simulation Study I and II, both indirect two-arm trial and three-arm trial surpass direct two-arm trial in power. Additionally, even if it is not for the consideration of power, choosing a two-arm indirect trial or three-arm trial rather than the direct two-arm trial can be attractive when treatment A is high-cost. By replacing some or all amounts of treatment A with an appropriate treatment B, we could reduce the cost and gain more power at the same time.

### How should we choose the optimal trial design

We learn that an indirect two-arm trial or three-arm trial can be more powerful than a direct two-arm trial for certain scenarios. How should we choose between those two candidates for the trial design? In our two sets of simulations, the indirect two-arm trial has larger power than the three-arm trial in Simulation Study I while it is the other direction in Simulation Study II. The two simulations are selected from the exploration result to present because each simulation exemplifies a potential scenario wherein one of the alternative trial designs exhibits maximal statistical power. In the exploration, we examined all possible permutations of treatments A and B in the existing network and calculated the power gain by using the two alternative trial designs based on the power formula. In the exploration, we observed that when $\sigma ^2_{AB, old}$ is smaller, the indirect two-arm trial design tends to outperform the three-arm trial design. This phenomenon is rationalized by the fact that diminished values of $\sigma ^2_{AB, old}$ denote heightened reliability in the existing evidence. Consequently, a judicious allocation of sample sizes to treatments Z and B becomes conducive, given the robustness of the estimation between treatments A and B. Moreover, the choice of total sample size *n* could also flip the choice of optimal trial design. In certain exploration scenarios, the optimal trial design changes from an indirect two-arm trial to a three-arm trial. This shift accentuates the potential utility of incorporating a direct A-to-Z comparison within the novel trial design, supplementing the indirect estimation between A and B obtained from the existing NMA. However, it is imperative to acknowledge that beyond the determinants of total sample size (*n*) and $\sigma ^2_{AB, old}$, a series of additional factors interplay to influence the variance of the estimated effect size between treatments A and Z, encompassing the associated risks linked to treatments A and B. The intricate interplay among these multifarious elements, as shown in the power formula, precludes the formulation of definitive guidance regarding threshold values that could singularly guide the selection process amongst these candidate trial designs. Therefore, in a real application, we advise the researcher to try our power formula and optimization to compare the optimal power that each trial design can reach to make the decision. It is general practice to specify values of the parameters (risks) in a statistical power calculation. These specific values in power calculation may come from estimation in previous studies, or can be based on experts’ opinions or research expectations. In case there is uncertainly in these parameter values, multiple calculations with a range of values can be performed and compared. Note that there are other factors to consider when choosing between an indirect two-arm trial and a three-arm trial given that they are both superior to a direct two-arm trial. For example, some clinical trials may be regulated by certain protocols, which may require having both treatments of the COI in the trial. Under that circumstance, an indirect two-arm trial is not feasible.

An alternative for determining the optimal trial design is to introduce adaptive design, which utilizes results accumulating in the trial to modify the trial’s course. Unlike the traditional approach of predefining a fixed trial design, adaptive design is more efficient, informative, and flexible [6]. We can apply a three-arm trial design as a start, then modify the ongoing trial by gradually adding the sample size to a certain group according to the interim results. Allocating more sample sizes to treatment group A gradually would mimic the performance characteristics of a two-arm trial. Increasing the sample sizes allocated to treatment group B brings about a behavior that closely resembles that of an indirect two-arm trial. In the context of adaptive design, data are repeatedly analyzed. Thus, we need to ensure that statistical inferences are correctly conducted with a controlled type I error rate. To facilitate this, we can draw from the established methods proposed by previous researchers. For example, Lu et al. [7] proposed a method to design the nested subpopulations, which maximize study power and keep the overall type I error rate under control. Leveraging their methods can help us to calculate the optimal sample size and the decision threshold for each sub-population, to provide a foundation for us to start the adaptive design. Moreover, some techniques used in trial monitoring, such as group sequential methods can also be leveraged to adaptive designs under certain conditions, as proved by Xuekui et al. [8]. In that way, each individual hypothesis can be tested at the full $\alpha$ level to give the study maximum power so that we could decide which question to answer by interim results and then answer the question with maximum power using all data.

### Limitations

There are some common limitations for all methodologies on NMA. The assumptions of NMA are required to be met, such as independence, exchangeability, transitivity and consistency. Those assumptions may not always be valid in real applications. Another limitation specific to this paper is that the possibility to gain more power from other types of trial design other than direct two-arm trials is network-dependent. There might not exist a suitable treatment B in the existing network that can bring more power by adding it to the trial design. For example, when all old treatments *i* have $p_i(1-p_i) \le p_A(1-p_A)$ where our COI is between A and a new treatment, it is impossible to gain more power theoretically by the other two trial designs.

### Future directions

We develop our proposed methodology under fixed-effect NMA because there is only one new trial involved with the new treatment where the between-study variation is unable to be evaluated. One future direction could be planning a series of new trials and applying the same concept of our methodology on top of the random-effect NMA, which may be more interesting to some researchers as random-effect NMA are also widely used. Another future direction could be to add the COI and explore how the formulas changes and how it guides our new trial planning in terms of the type of trial design, which is practical as well since some researchers are interested in exploring multiple comparisons in a new trial.

## Data Availability

We provide the R code and data we used in this paper in https://github.com/fangshuye/Three-arm-two-arm.

## Abbreviations

RCT: Randomized controlled trials
NMA: Network Meta-Analysis
COI: Comparison of Interest
BRD: Bovine Respiratory Disease
TULA: Tulathromycin
TRIM: Trimethoprim
CEFTS: Ceftiofur Sodium
